# Use of transcranial low-intensity focused ultrasound for targeted delivery of stem cell-derived exosomes to the brain

**DOI:** 10.1038/s41598-023-44785-1

**Published:** 2023-10-18

**Authors:** J. Haroon, K. Aboody, L. Flores, M. McDonald, K. Mahdavi, M. Zielinski, K. Jordan, E. Rindner, J. Surya, V. Venkatraman, V. Go-Stevens, G. Ngai, J. Lara, C. Hyde, S. Schafer, M. Schafer, A. Bystritsky, I. Nardi, T. Kuhn, D. Ross, S. Jordan

**Affiliations:** 1 The Regenesis Project, Santa Monica, CA USA; 2 Department of Stem Cell Biology & Regenerative Medicine, and Beckman Research Institute, City of Hope, Duarte, CA USA; 3 School of Biomedical Engineering, Science, and Health Systems, Drexel University, Philadelphia, USA; 4 Department of Psychiatry and Biobehavioral Sciences, University of California Los Angeles, Los Angeles, USA; 5 Kimera Labs Inc., Miramar, USA; 6 Department of Neurology, University of California Los Angeles, Los Angeles, USA

**Keywords:** Blood-brain barrier, Neuroscience, Stem cells in the nervous system

## Abstract

The blood–brain barrier (BBB) presents a significant challenge for targeted drug delivery. A proposed method to improve drug delivery across the BBB is focused ultrasound (fUS), which delivers ultrasound waves to a targeted location in the brain and is hypothesized to open the BBB. Furthermore, stem cell-derived exosomes have been suggested as a possible anti-inflammatory molecule that may have neural benefits, if able to pass the BBB. In the present study, transcranial low-intensity focused ultrasound (LIFU), without the use of intravenous microbubbles, was assessed for both (1) its ability to influence the BBB, as well as (2) its ability to increase the localization of intravenously administered small molecules to a specific region in the brain. In vivo rat studies were conducted with a rodent-customized 2 MHz LIFU probe (peak pressure = 1.5 MPa), and injection of labeled stem cell-derived exosomes. The results suggested that LIFU (without microbubbles) did not appear to open the BBB after exposure times of 20, 40, or 60 min; instead, there appeared to be an increase in transcytosis of the dextran tracer. Furthermore, the imaging results of the exosome study showed an increase in exosome localization in the right hippocampus following 60 min of targeted LIFU.

## Introduction

The blood–brain barrier (BBB) is a selective neurovascular barrier surrounding the brain that protects the central nervous system (CNS) from peripheral harmful toxins and serum factors^1^. Structural components of the BBB include endothelial cells, pericytes, astrocyte end feet, and microglia^1^. Together, these elements provide a robust barrier that regulates the influx and efflux of molecules into and out of the CNS^2^, but also presents a significant challenge for efficient drug delivery into the brain^3^.

Typically, drugs and molecules that efficiently cross the BBB are highly lipophilic molecules with a low molecular weight (< 400 Da) and a positive charge. Several approaches to circumvent the BBB for therapeutic applications have been tested with some success^3,4^; however, these methods may be highly invasive (direct brain injection), potentially harmful (ultrasound delivery with microbubbles) or ineffective at increasing therapeutic agent concentration within a target region (intranasal drug delivery). Additional methods for targeted cross-BBB drug delivery with improved safety and effectiveness are needed.

**Figure 1:**
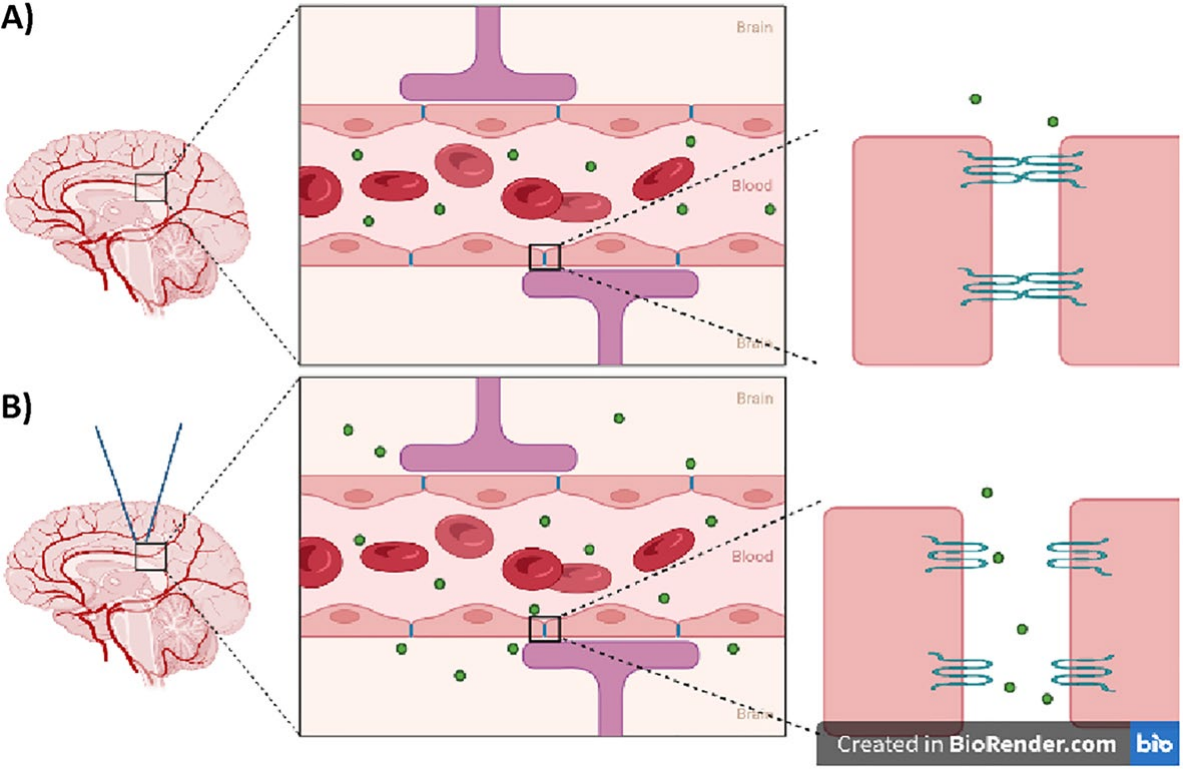
Illustration created in BioRender of the blood brain barrier. (**A**) Demonstrates how the blood brain barrier blocks the entrance of exosomes (green) into central nervous system. (**B**) The brain receiving low-intensity focused ultrasound allows the blood brain barrier releases exosomes from the blood stream.

Low intensity focused ultrasound (LIFU) is a well-established clinical technique for neurological diagnosis. Recently, LIFU has also been evaluated as a potentially non-invasive technique for selective delivery of small molecules, including drugs, normally unable to bypass the BBB. The use of focused ultrasound to modulate brain tissues can be traced back to 1942 when a preclinical study examined behavior changes after inducing brain lesions using High-Intensity Focused Ultrasound (HIFU)^5^. HIFU uses temperature and energy parameters extreme enough to produce brain lesions^6^ and has been utilized in humans to ablate tumors and for functional neurosurgery. In contrast, LIFU has been explored for its ability to modulate brain tissues without the ablative function of HIFU (Fig. 1).

The use of exosomes, which are small, lipid-bound extracellular nanovesicles shed by most cell types, has been posited as a potential strategic aid for therapeutic delivery across the BBB^7^. Stem cell-derived exosomes contain cytosolic proteins, messenger RNAs (mRNAs) and microRNAs (miRNAs)^8,9^, with biological activity determined by their anti-inflammatory, neuroregenerative and neuroprotective properties^10^. Neural Stem Cell (NSC)-derived exosomes, are enriched with miRNAs that modulate proximate and distant neuro-microenvironments, producing anti-inflammatory, neurogenic, and neurotrophic effects^11^. Therapeutic utility for these cell-free exosomes includes suppressing neuroinflammation, reducing axonal injury, and/or modulating differentiation of endogenous immature cells into neural-lineage brain cells. Exosomes have some capacity to cross the BBB via transcytosis^12–14^. The use of non-ablative LIFU to increase the transport of stem cell-derived exosomes across the BBB and enhance localization to targeted brain regions following IV administration has the potential to significantly augment their therapeutic CNS effects^4^. Exosome therapy should promote suppression of microglial inflammation and survival of endogenous neural progenitors, while preventing myelin loss, thus improving brain tissue plasticity and regeneration^15–17^.

Interestingly, the idea that exosomes could be directly localized to a particular brain region as specified by pre-treatment with transcranial non-ablative LIFU, has shown promising results. A preclinical study by Alptekin et al.^11^ explored whether the use of nanobubbles is required for LIFU to selectively deliver exosomes for stroke lesions. Nanobubbles (a contrast agent shown to enhance the acoustic effect of ultrasound) have been theorized as an agent that, when paired with LIFU, would create a sufficiently large enough disturbance to increase target area perfusion and permeability through the BBB^18^. Findings from Alptekin et al., confirmed that while LIFU with nanobubbles did improve exosome delivery across the BBB, the activity of the nanobubbles resulted in microbleeding and white matter damage. More importantly, however, results also indicated that LIFU stimulation administered independently, without nanobubbles, selectively increased the localization of exosomes to a specific targeted brain region^11,18^.

The Alptekin et al. study concluded that future research on targeted exosome delivery could be safely conducted using only LIFU, without the need for nanobubbles. Based on this conclusion, the initial purpose of our preclinical investigation was to assess changes in BBB permeability following treatment with LIFU (“Dextran Study”). Next, we investigated the quantitative differences in the localization of stem cell-derived exosomes with and without pre-treatment with LIFU (“Exosome Study”). No micro- or nanobubbles were used in either study.

The present studies utilized 2 MHz LIFU. The measured peak rarefactional pressure (water value) was 1.5 MPa, with a calculated Mechanical Index of 0.76, is below the expected threshold for cavitation. The pulse sequence (100 Hz PRF, 0.06 ms pulse duration, 0.6% duty cycle) would preclude tissue heating effects (Thermal Index Cranial of 0.19). As such, 2 MHz LIFU was selected as it is a frequency comparable to current diagnostic ultrasound use with extensive history of proven safety in humans.

## Results

### Dextran study: focused ultrasound effect on BBB endothelium, following intravenous injection of dextran

The dextran study assessed the effects on the BBB endothelium with and without LIFU and examined if there was any BBB disruption dependent on the duration of LIFU exposure. Four female Wistar rats (age 90 days) were anesthetized by intraperitoneal (IP) injection of a Ketamine/Xylazine/Acepromazine cocktail and placed in a stereotactic apparatus for treatment. To assess for BBB changes, a dextran solution containing antibodies for FITC10 and RHO70 was injected into the tail vein of each rat immediately following either 0, 20, 40, or 60 min of LIFU to the right hippocampus. Treatment conditions for each rat are listed in Supplementary Table 1.

**Figure 2:**
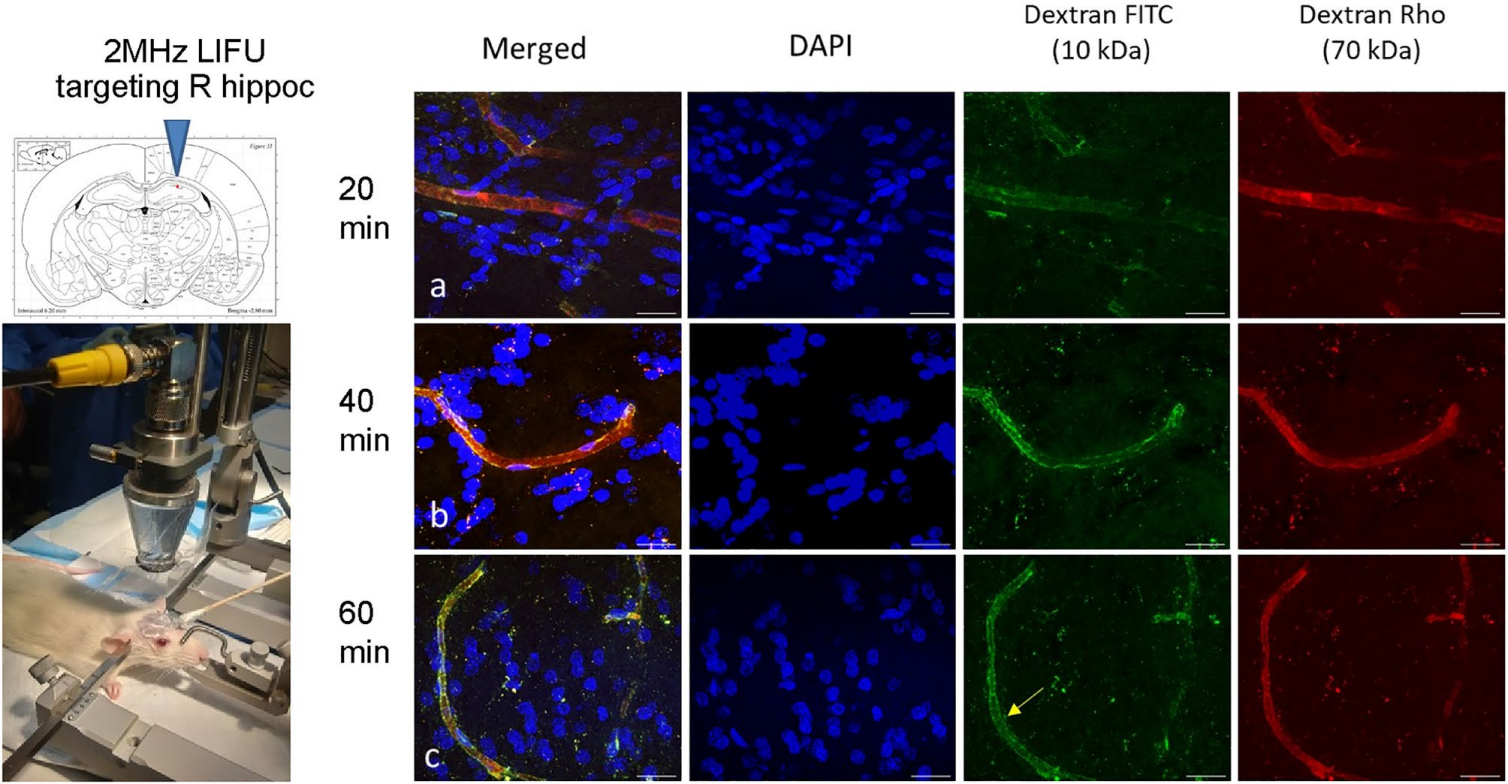
The effect of LIFU on BBB integrity shows increased leakage of dextran tracer in the right hippocampus, treated with LIFU. (Magnification at 63×, oil) (Photo and schematic of LIFU focused on R hippocampus). Right hippocampus z-stack images of a rat brain following 20, 40, and 60 min LIFU. At 60 min, leakage can be observed congregating in the endothelial cells and possibly outside the cell on the right side of the brain (yellow arrow). Scale bar at 20 µm.

No notable changes were seen in the BBB of the rat receiving 20 min of LIFU (Fig. 2a). Minimal changes were seen after 40 min of LIFU (Fig. 2b): the images show light perfusion of dextran and no significant leakage outside of the capillaries. However, for the rat exposed to 60 min of LIFU (Fig. 2c; Rat #4), dextran signal (green) appears to increase adhesion and transcytosis across endothelial cells, with a suggestion of dextran molecules outside the cells compared to 20 min. There were no pathological changes to endothelial structure to suggest edema or bleeding in the rat that received 60 min of LIFU (Supplementary Fig. 1).

### Exosome study: IV delivered exosome BBB penetration with and without LIFU

Having observed the largest effect on BBB permeability following 60 min of LIFU in the 60-min session of the Dextran Study, we next investigated the extent to which exosome accumulation would increase in the LIFU-targeted brain region. Four male Lewis rats (6 months old) were used for this study. Three rats received 60 min of LIFU treatment targeted at the right hippocampus, with the left hippocampus as an internal control, and the fourth rat was used as a no LIFU control (See Supplementary Table 2 for treatment conditions).

**Figure 3:**
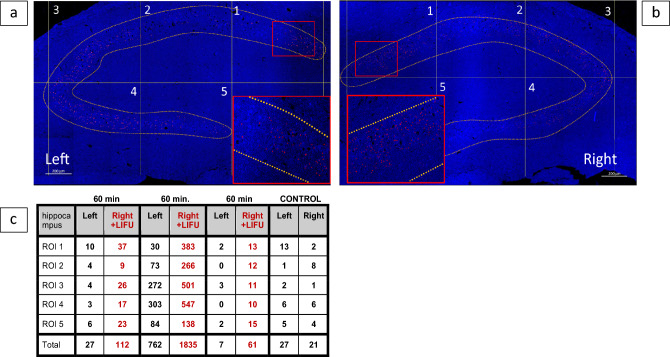
LIFU to Right Brain Hippocampus Results in Increased IV Exosome Localization. Left (**a**) and right (**b**) tissue section images of rat receiving 60 min LIFU to right hippocampus and IV exosomes (1.5 × 10^8^). Five regions of interest (ROIs) were counted along the CA1, CA2, CA3 gray matter track using Reconstruct (www.synapses.bu.edu) (Scale bar 200 µm). Autotracing parameters for hue, brightness, and saturation were set for Alexa fluor 647 stained exosomes (red). Table of exosome counts (**c**) for 3 treated rats (varying LIFU parameters), and 1 control (no LIFU). In all cases, exosome counts were significantly higher in LIFU right hippocampus.

**Figure 4:**
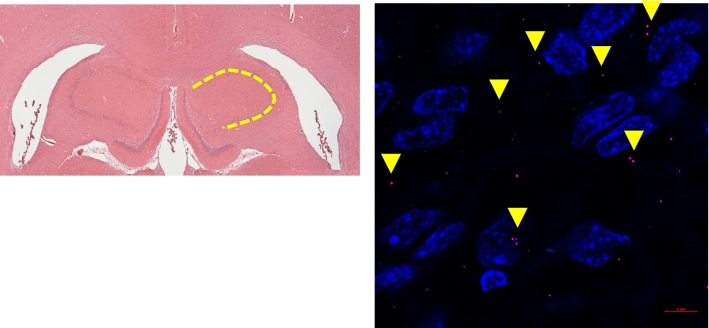
Region of exosome counts (Left) Location of area quantified. Exosomes counted along the CA1, CA2, and CA3 gray matter track (highlighted in yellow). (Right) Confocal imaging of Renilla luciferase antibody with Alexa fluor 647 staining of exosomes (arrows) present in the hippocampus (right side) of the rat brain following 60 min of LIFU (Magnification, 63× oil. Scale bar at 5 µm).

In Rat #6, exosome concentration was elevated in the targeted right hippocampus when compared with the left hippocampus internal control (Figs. 3, 4). A limited statistical analysis of the exosome counts (Supplementary Table 3, Supplementary Fig. 2) showed a statistically significantly higher number of counted exosomes in the LIFU-targeted right hemisphere versus the control left hemisphere (*U* = − 3.27, mean difference = 129.67, *p* < 0.001). When comparing the exosome count in the right hemisphere of Rat #6 to the right no LIFU control animal (Rat #8) there is also a strong significant difference (*U* = − 2.61, mean difference = 362.8, *p* = 0.008).

## Discussion

The delivery of biological agents (e.g., stem cells, exosomes) to the brain has been the subject of numerous studies aimed at the reversal of a variety of conditions, including aging^19,20^, stroke^21,22^, and degenerative disease^23,24^. These efforts have either used repetitive and prolonged administration or invasive techniques that may not be suitable for routine deployment in a clinical setting. The present study demonstrates the possibility of delivering biologicals with the help of an ultrasound device, which has decades of safety data in humans. Current ultrasound devices paired with optical tracking, could be deployed safely, accessibly, and inexpensively in a clinical setting. The preliminary data in this report warrants further study and supports the feasibility of pursuing applications in humans.

Our first goal was to determine whether LIFU alone (without microbubbles) had a time-dependent effect on the BBB endothelium, as evaluated using a dextran tracer. Our second goal was to use the same low-intensity, diagnostic-level (2 MHz) LIFU, to determine whether targeted ultrasound could increase the concentration of IV exosomes to a specific brain location.

The imaging results of the dextran study suggest that LIFU did not damage the BBB at 20-, 40-, or 60-min exposure times. There was no marked penetration of tracer into the brain and no obvious evidence of edema or bleeding. Increased dextran concentration in the endothelium suggests an increase in transcytosis in a time-dependent relation to LIFU treatment time^25^.

The imaging results of the exosome study showed an increased concentration of exosome localization to the right hippocampus, following 60 min of LIFU focused to that target. This is supported by the lack of exosome concentration seen in the control rats which received IV exosomes alone, without focused ultrasound. Rat #6, which received IV exosomes after 60 min of right brain targeted LIFU, shows a significantly increased exosome concentration in the treated (right) hippocampus control compared to the control (left) hippocampus. The distribution of exosomes also appears to have preferentially localized to the grey matter track of the hippocampus; understanding this mechanism will require future studies will larger sample sizes to explore reproducibility.

Notably, however, the left untreated hippocampus of Rat #6 shows an increased concentration of exosomes relative to the no LIFU control (Rat #8). This may be explained by the small size of the rat head resulting in increased refraction of the ultrasound beam. Further adjustments are being made to more tightly focus the LIFU beam for rodent applications, but this is more easily accomplished in human applications, with larger regions of interest. Furthermore, the right hippocampus of Rat #6 showed a significantly elevated total exosome count when compared to Rat #5 and Rat #7 (count = 1835, 112, 61, respectively). The reasons for this are not fully understood, though exosome extravasation in the tail vein may be one possibility.

This study reports the first evidence that LIFU administered at 2 MHz—the frequency safely used in humans at the diagnostic level with Transcranial Doppler technology—targeted at a precise brain region demonstrates increased concentration of IV exosomes to the targeted location. The ultrasound exposure was designed to have no cavitational or thermal effect. Our hypothesis is that there is a mechanical effect, possibly from acoustic radiation force or a pressure differential across the cellular membranes that is causing the results noted. We observed no evidence of tissue damage, micro-bleeding, or other untoward effects that may be seen in applications of high-intensity focused ultrasound (HIFU), though further studies must be conducted to confirm safety and translatability.

If confirmed to be safe, this technique could therefore be a potentially safe, affordable, and effectively deployable technique in patient clinics. This present study was conducted using LIFU at intensities below the FDA derated maximum of I_SPTA.3_ of 720 mw/cm^2^, which would indicate that similar exposures would be possible in the clinic without undue regulatory or safety issues. Transcranial Doppler (TCD) systems operate at 2 MHz, and are presently used continuously for hours to monitor cerebral bloodflow during or after surgeries^26,27^. TCD systems are applied through the “temporal window” where the skull is relatively thin and uniform, allowing for relatively good acoustic penetration and clinical results. Clinically, a larger volume of the brain would be targeted, and therefore a lower frequency could potentially be used. For instance, at 650 kHz, a 4 mm diameter beam width is possible, deep within the skull.

Limitations to this study include sample population and size, and future studies with larger sample sizes and uniform subject characteristics (sex, age, etc.) are needed to support the validity and reproducibility of these results. However, if further explored, the use of LIFU for targeted delivery of IV stem cell-derived exosomes and other small molecules may significantly enhance therapeutic efficacy to degenerative and inflammatory CNS disorders.

## Conclusion

This study highlights the exciting potential of low-intensity focused ultrasound (LIFU) for precise delivery of therapeutic biological agents (such as exosomes produced from stem cells) to specific brain regions, without compromising the blood–brain barrier. The results support the use of LIFU as a safe, practical, and affordable technique for improving therapeutic efficacy in inflammatory and degenerative CNS illnesses. Targeted drug delivery by LIFU has the potential to revolutionize therapeutic therapies and enhance patient outcomes, but more research with larger sample sizes is required to validate and reproduce these findings.

## Methods

### Focused ultrasound probe for rat studies

For these experiments, an ultrasound exposure system was custom designed to insonate the right hippocampus of the rat brain with sufficient localization to leave the left hippocampus minimally unexposed, allowing the unexposed side to serve as a control. Acoustic simulations were conducted to examine the effects of transducer diameter, frequency, and focal length. Based on those results, the 2 MHz, 25 mm diameter, 48 mm nominal focal depth transducer was chosen as a compromise between competing requirements. Extensive beam mapping was conducted to verify the simulation (See Supplementary Figs. 3 and 4). Calibrated hydrophone measurements were conducted to establish the peak pressure levels. Higher ultrasound frequencies permit better focusing power; however, attenuation also increases with frequency, and the goal was to minimize attenuation through the rat skull. An operating frequency of 2 MHz was chosen as a compromise between these competing requirements. Other parameters included a Pulse Repetition Frequency (PRF) period of 10.00 ms, a Duty Cycle of 0.6%, and Peak Rarefractional Pressure (p_r_) of 1.5 MPa (Supplementary Table 4). Commercial single-element focused ultrasound transducers (Olympus NDT and Technisonic, 25 mm diameter, 48 mm focal depth) were obtained and fully characterized in terms of focal zone extent and dimension^28,29^. The beam pattern measurements indicate that the acoustic intensity away from the main beam (i.e. the sidelobes) is significantly reduced (less than 2% of the peak intensity) and the goal of minimal exposure of the ipsilateral hippocampus was met.

The low intensity and low duty cycle exposure minimized any potential heating effects. The peak pressure, intensity, and total acoustic power are well below diagnostic ultrasound levels and approved for continuous use. As a check, the Thermal Indices, both TI Cranial (at the bone surface) and TI Soft Tissue (tissue near the beam entrance place and focus) were calculated and found to be below 0.2. Since the brain tissue is well perfused, it is unlikely that there was any thermal effect due to ultrasound exposure. The Mechanical Index was calculated to be 0.77, which would indicate that there is no potential for cavitation.

To position the focal location at the correct depth within the rat skull, a water-filled standoff was designed to provide a 42 mm offset, with an acoustically transparent membrane at the exit location. The ultrasound beam was 2.1 mm wide and extended 11 mm out from the exit plane.

The low intensity and low duty cycle exposure minimized any potential heating effects. The peak pressure, intensity, and total acoustic power are well below diagnostic ultrasound levels and approved for continuous use. The time average intensity I_SPTA.0_ is 668 mw/cm^2^, or a derated intensity I_SPTA.3_ of 632 mW/cm^2^, which is below the FDA diagnostic limit of I_SPTA.3_ of 720 mw/cm^2^. The derivation of the Thermal Indices for stationary beams were based on circular beams such those used here and assumed steady state (long duration) exposure; therefore, the calculations are relevant to estimation of potential heating. The Thermal Index Cranial (at the bone surface) assumes complete absorption of ultrasound energy by the skull but does provide a worst-case value of 0.19. Further, a calculation of the Thermal Index, Soft Tissue (tissue near the beam entrance place and focus) for small non-scanning beams results in a value of 0.15. This would be an estimate of the maximum heating assuming no bone attenuation. These two values therefore represent opposite worst case conditions, (i.e., total absorption by the skull, and no skull absorption). Since both are within the diurnal variation of ~ 1 °C in rodents (and humans)^30^, the temperature effect is minimal. Further, since brain tissue is well perfused, it is unlikely that there was any thermal effect due to ultrasound exposure. The Mechanical Index was calculated to be 0.77, which would indicate that there is no potential for cavitation.

### Dextran preparation

To assess the BBB integrity, two fluorescent dextran tracers were used to visualize BBB permeability. One dextran had a small molecular weight (FITC—10 kDa) to visualize extravesicular particle movement. The other dextran had a large molecular weight (Rhodamine—70 kDa) to visualize the luminal edge.

A FITC dextran solution was prepared by dissolving fluorescein isothiocyanate-dextran (FITC10, Sigma-Aldrich, St. Louis, MO, USA; FD10S) in sterile saline at 10 mg/ml, then filtered using a 0.22 µm filter for use at 2.86 µl/g for each rat. Next, a rhodamine dextran solution was prepared by dissolving rhodamine B isothiocyanate-dextran (RHO70, Sigma-Aldrich, St. Louis, MO, USA; 283924) in sterile saline at 10 mg/ml, then filtered using a 0.22 µm filter at enough volume to inject 2.86 µl/g for each rat. Once FITC10- and RHO70-dextrans were in solution, the two fluorescent solutions were mixed (1:1) then filtered a final time using a 0.22 µm filter.

### Exosome preparation

A 5 ml vial of XoGlo Pro Extravesicles (Evs) (Kimera Labs, Miramar Fl) was incubated with 10 µg of a biotinylated CD9 antibody (Abcam, ab28094), and 10 µg of a biotinylated CD63 antibody (Abcam, ab134331) at room temperature for 3 h with gentle rocking. After this incubation, 7.5 µg of a Streptavidin-Renilla Luciferase fusion protein (Ray Biotech, 230-00052-15) was added to the XoGlo Pro vial, and the Evs were incubated for an additional hour at room temperature with gentle rocking. The Evs were then dialyzed in a 100 kDa cut-off dialysis tube (Thermo, 88532) at 4 °C against 1X PBS for 16 h with 3 subsequent changes of PBS every 6 h. Subsequent labeled XoGlo Pro Evs were then used for further experiments.

### Animal studies

All animal experimental procedures were conducted in compliance with the Guide for the Care and Use of Laboratory Animals of the NIH, the ARRIVE guidelines, and were also approved by the Institutional Animal Care and Use Committee of the Beckman Research Institute, City of Hope (IACUC 20037). Animals were housed in groups of 2 in large, ventilated cages with ad libitum food and water under a 12/12-h light/dark (lights on at 07:00) cycle in a room with controlled temperature (22 ± 2 °C) and humidity (55 ± 5%).

#### Dextran study

This study was performed on 4 female Wistar rats (age 90 days) during their 12-h light cycle. Rats were anesthetized using an intraperitoneal (IP) cocktail of Ketamine/Xylazine/Acepromazine at a concentration of 0.1 μl/100 g. Following anesthesia, the rat’s head was mounted on the stereotaxic apparatus (Stoelting Co., USA) with ear and nose bars. Ultrasound transmission gel was applied to the shaved head and the ultrasound probe was mounted and directed toward the targeted area of the brain, the right hippocampus (6.2 mm rostral from interaural, 2.00 mm lateral right) (Fig. 3a). Each LIFU session was performed at a set parameter (Supplementary Table 4) for each duration period (Supplementary Table 1). Immediately following LIFU, treated rats received a 1 mL intravenous injection of labeled dextran solution (2.86 µl/g per rat) into a pre-warmed tail vein which was allowed to circulate for 45 min. Rats were then euthanized using CO_2_ gas according to AVMA Guidelines for Euthanasia of Animals. Brains were then dissected out. Harvested brains were immediately placed into 4% paraformaldehyde for fixing. After 48 h, brains were placed into a 30% sucrose solution for cryoprotection and further tissue processing.

#### Exosome study

This study was performed on 4 male Lewis rats (6 months old) during their 12-h light cycle. Rats were anesthetized using an intraperitoneal (IP) cocktail of Ketamine/Xylazine/Acepromazine at a concentration of 0.1 μl/100 g. Following anesthesia, the rat’s head was mounted on the stereotaxic apparatus (Stoelting Co., USA) with ear and nose bars. Transmission gel was applied to the shaved head and the ultrasound probe mounted and directed toward the targeted, the right hippocampus (6.2 mm rostral from interaural, 2.00 mm lateral right) (Fig. 3a).

Each LIFU session was performed at a set parameter (Supplementary Table 4) for a total of 60 min). Immediately after LIFU, 1 mL of Rluc-labeled exosomes (1.5 × 10^8^/1 ml per rat) was injected into a pre-warmed tail vein and allowed to circulate for 90 min. Rats were then euthanized using CO_2_ gas according to AVMA Guidelines for Euthanasia of Animals. Brains were then dissected out. Harvested brains were immediately placed into 4% paraformaldehyde for fixing. After 48 h, brains were placed into a 30% sucrose solution for cryoprotection and further tissue processing.

### Tissue preparation, confocal microscopy, and image analyses

#### Dextran study

To histologically assess the brain tissue and BBB integrity, fixed brains were mounted, serial cryosectioned horizontally at 30 µm, and collected onto slides. Sections were selected to ensure that different levels of the brain were represented. Slides were then coverslipped with ProLong Gold Antifade Mountant with DAPI (ThermoFisher Scientific, Waltham, MA, USA; P36931) and allowed to cure at room temperature in the dark for 24 h. Slides were then kept at − 20 °C for long-term storage. Confocal imaging and image analysis were employed. For each cryosection, the hippocampus was located and imaged using a Zeiss 880 Airyscan confocal microscope (Zeiss, Oberkochen, Germany) with 488, 561, and UV diode lasers to visualize FITC10, RHO70, and DAPI, respectively. For each section, two images were acquired systematically throughout the hippocampus to acquire the same region for each rat. Each site was imaged by z-stack using a 63 × 1.4 N.A. oil objective lens with a resolution of 2048 × 2048 pixels.

To analyze images, images were opened in FIJI with split channels to visualize each channel individually and then merged. Images were then assessed for signs of FITC penetration and co-localization of FITC10 and RHO70 dextran molecules.

#### Exosome study

To assess exosome localization following LIFU, fixed brains were mounted in OCT, cryosectioned coronally at 20 µm, and collected onto slides. Slides from each level were blocked using a preformulated solution comprised of 5% animal source protein (ThermoScientific SuperBlock Blocking Buffer, Chicago, IL, #37515) for 1 h, followed by incubation with primary antibody Anti-Rluc (Abcam, ab187338) overnight at 1:1000. Sections were then washed in PBS for 10 min and incubated with the corresponding secondary antibody at 37 °C for 1 h, Alexa fluor 647 chicken anti-rabbit (Invitrogen, 1:400). Slides were then coverslipped with ProLong Gold Antifade Mountant with DAPI (ThermoFisher Scientific, Waltham, MA, USA; P36931), and allowed to cure at room temperature in the dark for 24 h. All tiled images were then captured using Zeiss LSM 900 Airyscan confocal microscope. (Light Microscopy Core, City of Hope, Duarte CA). Exosomes were counted via 3D Reconstruct. Representative Alexa fluor 647 stained exosomes (red) were selected to establish the acceptable range for the parameters (hue, brightness, and saturation), with which Autotracing (wildfire tool) of the program generated automated counts. A Mann–Whitney U test was conducted to compare exosome count in the right hemisphere of the three animals (Rat #5, 6, 7) that received LIFU to the exosome count in the right hemisphere of the control animal that did not receive LIFU (Rat #8). A follow up analysis compared the right hemisphere exosome count between the animal with the most optimal exosome count following LIFU (Rat #6), to the right hemisphere exosome count in the control animal (Rat #8).

### Supplementary Information

Supplementary Information.

## Data Availability

The data that support the findings of this study are available from the authors upon reasonable request by contacting lead authors Dr. Karen Aboody, M.D. (Kaboody@coh.org) or Jonathan Haroon (Jharoon@theneuroassociates.com).
