# Supplementary material for: Use of transcranial low-intensity focused ultrasound for targeted delivery of stem cell-derived exosomes to the brain
Source: Sci Rep. 2023 Oct 18;13:17707. doi: 10.1038/s41598-023-44785-1 (PMC10584845; doi:10.1038/s41598-023-44785-1)
Supplement: Supplementary file 1 — Supplementary Information. [file 41598_2023_44785_MOESM1_ESM.docx]

**Supplementary Tables and Figures**

**Supplementary Table 1. Treatment conditions for dextran study**

| **Animal #** | **Sex** | **Focused Ultrasound** | **Duration** | **Tracer** |
| --- | --- | --- | --- | --- |
| 1 | F | - | None | Dextran |
| 2 | F | + | 20 minutes | Dextran |
| 3 | F | + | 40 minutes | Dextran |
| 4 | F | + | 60 minutes | Dextran |

**Supplementary Table 2. Treatment conditions for exosome study**

| **Animal #** | **Sex** | **Focused Ultrasound** | **Duration** | **Exosome Injection** |
| --- | --- | --- | --- | --- |
| 5 | M | + | 60 minutes | IV (1.5 x10^8^/1 mL) MSC.exo.Rluc |
| 6 | M | + | 60 minutes | IV (1.5 x10^8^/1 mL) MSC.exo.Rluc |
| 7 | M | + | 60 minutes | IV (1.5 x10^8^/1 mL) MSC.exo.Rluc |
| 8 | M | - | N/A | IV (1.5 x10^8^/1 mL) MSC.exo.Rluc |

**Supplementary Table 3. Exosome counts, sectioned in each rat**

|  | Rat 5 | | Rat 6 | | Rat 7 | | Rat 8 (control) | |
| --- | --- | --- | --- | --- | --- | --- | --- | --- |
|  | Left | Right | Left | Right | Left | Right | Left | Right |
| ROI 1 | 10 | 37 | 30 | 383 | 2 | 13 | 13 | 2 |
| ROI 2 | 4 | 9 | 73 | 266 | 0 | 12 | 1 | 8 |
| ROI 3 | 4 | 26 | 272 | 501 | 3 | 11 | 2 | 1 |
| ROI 4 | 3 | 17 | 303 | 547 | 0 | 10 | 6 | 6 |
| ROI 5 | 6 | 23 | 84 | 138 | 2 | 15 | 5 | 4 |
| Total | 27 | 112 | 762 | 1835 | 7 | 61 | 27 | 21 |

**Supplementary Table 4. Parameters of LIFU treatment**

| **Pulse Length** | 0.06 ms |
| --- | --- |
| **PRF Period** | 10.00 ms |
| **Duration** | 3600 seconds |
| **Duty Cycle** | 0.6% |
| **Frequency** | 2 MHz |
| **Peak Rarefactional Pressure (pr)** | 1.5 MPa |
| **Transducer diameter** | 25 mm |
| **Transducer focal distance** | 48 mm |
| **Water-filled cone depth** | 42 mm |
| **Minimum Beam Focal Width** | 2.1 mm |
| **Beam Focal Length Extent, pressure*** | 11 mm |
| **Beam Focal Length Extent, width**** | 8.0 mm |

* This is the depth over which the pressure remains within 50% of the peak rarefactional pressure, starting from the exit plane. The pressure drops significantly after this depth.

** This is the depth over which the beam width remains within 10% (0.2mm) of the minimum width, starting from the exit plane. The beam widens significantly after this depth.

**Supplementary Table 5. Detailed Protocol of Staining Procedures**

| **Action** | **Time** | **Concentration** | **Company and Catalogue #** |
| --- | --- | --- | --- |
| Slides were removed from the -20 ° C freezer and bake at in a dry oven at 55ºC | 20 Minutes | N/A | N/A |
| Slides were then post fixed in 4% Parafomaldehyed | 10 minutes | 4% | 4% Parafomaldehye in PBS: Boston Bio Products Inc. Cat: BM-220 |
| Rinsed 3 times | 10 minutes each rinse | 1 x PBS (diluted from 10x) | 10 x PBS pH 7.4: Boston Bio Products Inc. Cat: B M -155 |
| Incubated all slides in 100 % blocking solution: SuperBlock in PBS | 1 hour | N/A | Thermo Scientific Cat: 37515 |
| Incubated at 4ºc in Primary antibody diluted in (5 % SuperBlock, 1 % Triton X-100 in 1 x PBS) | Overnight | 1 : 1000 | Abcam Cat: ab187338 RB P Ab to Renilla (Thermo Scientific Cat: 37515, Sigma: T9284, 10 x PBS pH 7.4: Boston Bio Products Inc. Cat: B M -155 diluted to 1 x PBS) |
| The next day, rinse the slides 4 times | 10 minutes each | 1 x PBS (diluted from 10 x) | 10 x PBS pH 7.4: Boston Bio Products Inc. Cat: B M -155 |
| Incubated with Secondary antibody diluted in (5 % SuperBlock, 1 % Triton X-1 0 0 in 1 x PBS) | 1 hour | 1 : 400 | Life Technologies Cat: A21443 Alexa Fluor. 647 Chicken anti Rabbit (Thermo Scientific Cat: 37515, Sigma: T9284, 10 x PBS pH 7.4: Boston Bio Products Inc. Cat: B M -155 diluted to 1 x PBS) |
| Rinsed the slides 4 times | 10 minutes each | 1 x PBS (diluted from 1 0 x) | 1 0 x PBS pH 7. 4 : Boston Bio Products Inc. Cat: B M -1 5 5 |
| Mounted with Dapi included Mounting Medium | Dry Covered 24 hours before imaging | N/A | Invitrogen ProLong Gold antifade Mountant Cat: P 3 6 9 3 4 |

**Supplementary Figure 1. H&E staining right hippocampus**


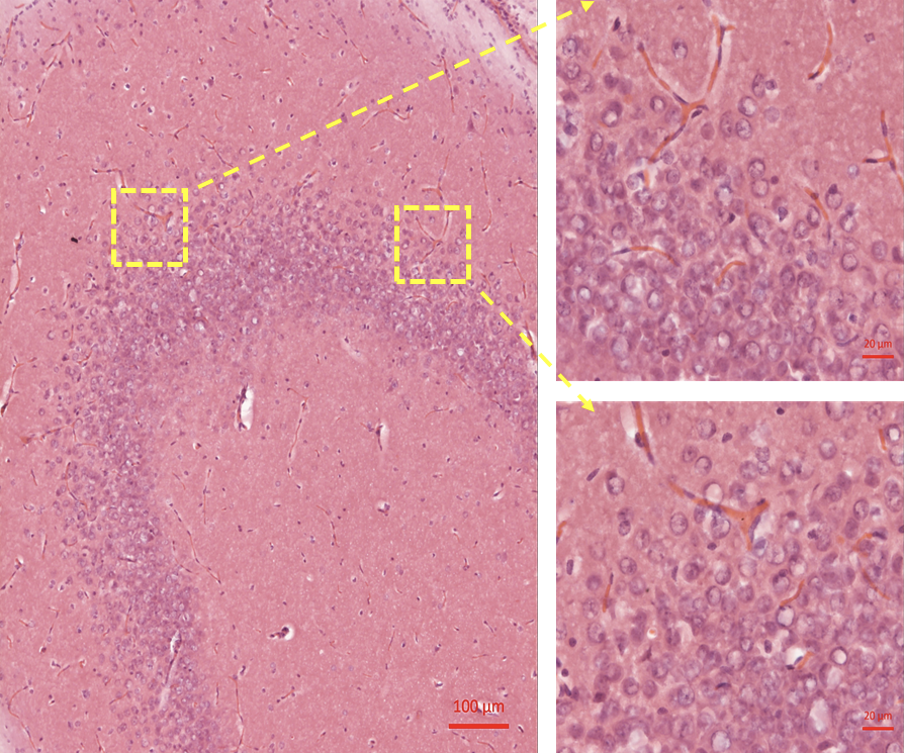


**Supplementary Fig. 1.** H&E staining of 60 min LIFU (right hippocampus) shows there were no pathological changes to the structure of the endothelium to suggest edema or bleeding. (40x, 20x).

**Supplementary Figure 2: Graph depiction of exosome counts**


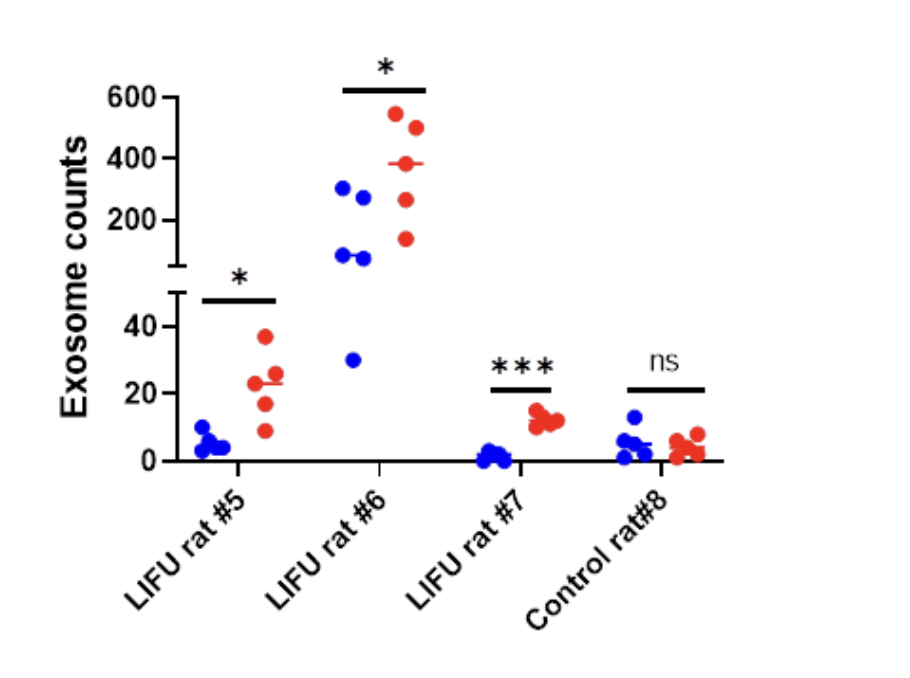


**Supplementary Fig. 2: Graph depiction of exosome counts, sectioned in each rat** (counts derived from Supplementary Table 3).

**Supplementary Figure 3. Lateral beam intensity**

**Supplementary Fig. 3.** Measurement of the lateral beam profile of the transducer, at the focal plane. Main lobe beam width 2.1mm; Sidelobe levels between -18dB to 19dB, which correspond to an intensity level of 1.5% relative to the main beam. Beam is circularly symmetrical, so the X and Y beam cross sections were averaged to produce this composite profile.

**Supplementary Figure 4. Ultrasound Beam Distribution**


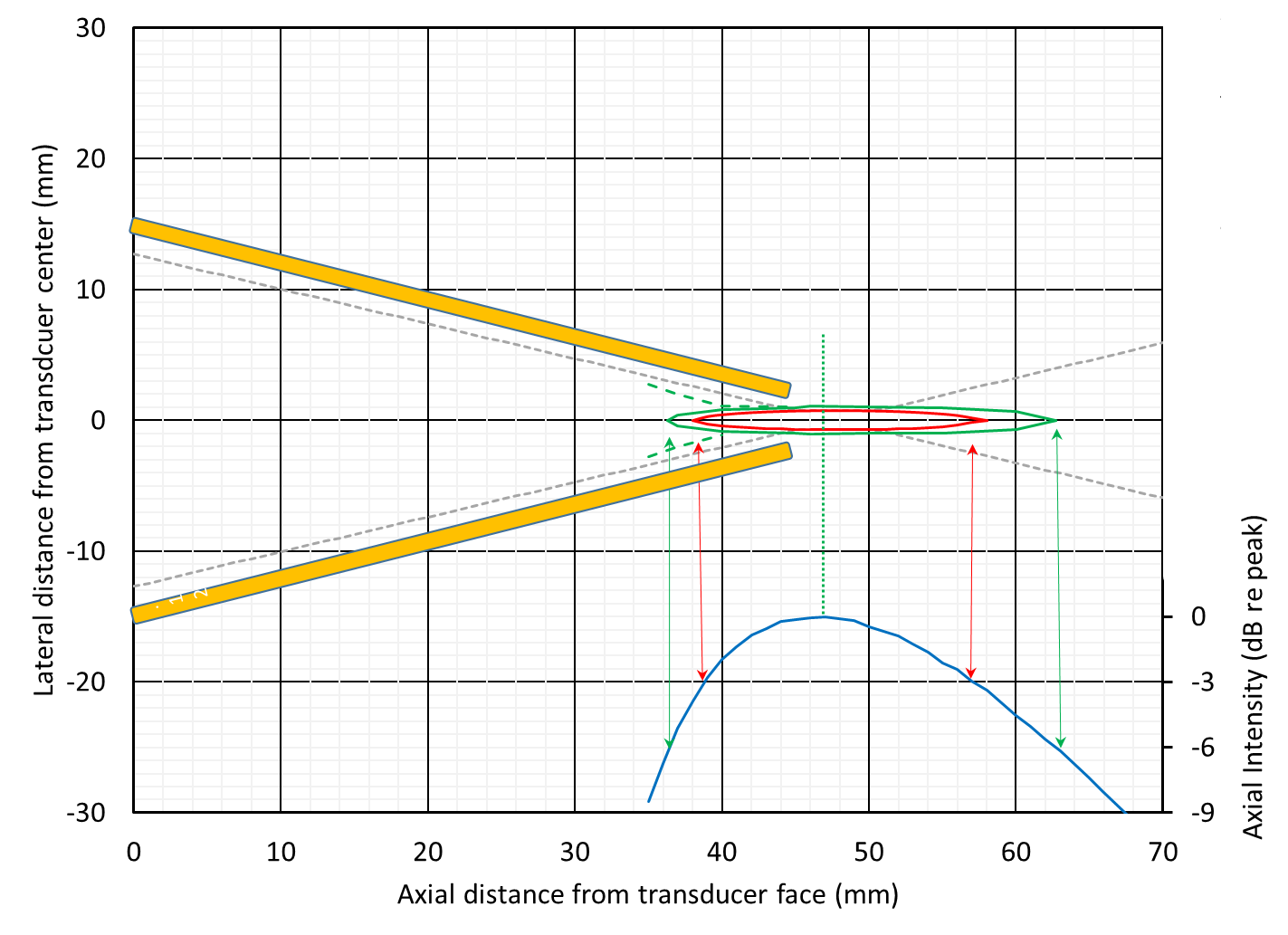


**Supplementary Fig. 4.** The circular transducer is located at the left edge of the graph, centered on 0, and aimed to the right. The yellow bars represent a cross section of the water filled coupling cone. The red and green ovals represent the -3dB and -6dB contours with respect to the peak respectively. The blue line represents the measured axial intensity (right hand axis) in dB relative to the peak. Measurements were performed before the water filled cone was attached, and then confirmed afterwards at the focal plane. The red and green arrows show the correspondence between the axial profile and the contour plots. The focal plane is shown by the dotted vertical line. The dashed lines are the theoretical geometrical beam extent.
